# Temporal summation in human peripheral axons when stimulated transcutaneously with a 10‐kHz waveform

**DOI:** 10.1113/EP092659

**Published:** 2025-10-15

**Authors:** Billy L. Luu, Harrison T. Finn, Terry Trinh, Simon C. Gandevia, Martin E. Héroux, Jane E. Butler

**Affiliations:** ^1^ Spinal Cord Injury Research Centre Neuroscience Research Australia Randwick New South Wales Australia; ^2^ School of Biomedical Sciences, Faculty of Medicine and Health University of New South Wales Sydney New South Wales Australia; ^3^ School of Clinical Medicine, Faculty of Medicine and Health University of New South Wales Sydney New South Wales Australia; ^4^ Prince of Wales Hospital Randwick New South Wales Australia

**Keywords:** axonal membrane, burst‐modulated currents, Gildemeister, subthreshold depolarisation

## Abstract

Transcutaneous spinal cord stimulation, as used for rehabilitation of impaired motor function after spinal cord injury, often involves a 10‐kHz waveform modulated to produce repetitive bursts of stimulation. Kilohertz‐frequency waveforms may facilitate the summation of subthreshold depolarisations, but the optimal burst duration for nerve stimulation has not been systematically investigated. In 11 adults, the ulnar nerve was stimulated transcutaneously with a 10‐kHz waveform that contained 1, 2, 4, 6, 8 or 10 pulses, in random order. Compound muscle action potentials (CMAPs) and sensory nerve action potentials (SNAPs) were measured from motor threshold up to the maximal CMAP (*M*_max_). The efficacy of each waveform was determined at *M*_max_ as CMAP amplitude divided by total phase charge. For CMAPs and SNAPs, increasing the number of pulses shifted the stimulus–response curves to the left for current and to the right for total charge. Accordingly, an increase in the number of pulses decreased the current but increased the total charge at sensory and motor thresholds and *M*_max_. Efficacy decreased as the number of pulses increased. Onset latencies were delayed for waveforms with six or more pulses compared to a single pulse. These findings provide evidence of the summation of subthreshold depolarisations in sensory and motor axons in humans. However, the optimal number of pulses for summation remains unclear due to the opposing changes in current and total charge. It is clear, though, that more than six pulses is suboptimal, as there were no further decreases in threshold current while total charge continued to increase.

## Abstract (highlights)

- **What is the central question of this study?**
  Does electrical stimulation with kilohertz‐frequency waveforms facilitate the summation of subthreshold depolarisations of the nerve fibre membrane in peripheral sensory and motor axons?
- **What is the main finding and its importance?**
  Temporal summation with a 10‐kHz waveform occurs over a short time course in peripheral axons, and bursts of stimulation of long duration are not optimal for activating sensory and motor axons.

## 1 INTRODUCTION

Transcutaneous electrical stimulation with a kilohertz‐frequency waveform has been investigated in many studies for strength training (Selkowitz, 1989) and for the rehabilitation of impaired motor function after spinal cord injury (Rehman et al., 2023). Along with a misconception of reduced discomfort (Dalrymple et al., 2023; Luu et al., 2024; Manson et al., 2020), it is thought that kilohertz‐frequency waveforms facilitate the summation of subthreshold depolarisations of the nerve fibre membrane, as the time between successive pulses is less than the time for ion concentrations to return to resting levels (Ward & Chuen, 2009; Ward & Robertson, 2001). As a result, the stimulus intensity required to generate an action potential will decrease as the number of pulses in the stimulus waveform increases. This process of temporal summation was first proposed by Gildemeister (1944) to explain the sensations produced by kilohertz‐frequency, alternating current stimulation (see Ward, 2009; Ward & Robertson, 2001).

Ward & Robertson (2001) have since provided support for a Gildemeister effect in motor axons innervating the wrist extensor muscles. They showed that stimulation with 10‐ms bursts of a range of sinusoidal waveforms (1–25 kHz) resulted in lower motor thresholds than single sinewaves with the same periods when both were repeated at 50 Hz. These repetitive bursts of stimulation, otherwise known as burst‐modulated currents, were also shown to have similar motor thresholds as continuous stimulation, indicating a potential limit to the summative effects of kilohertz‐frequency waveforms. Later studies by Ward and colleagues determined that the lowest motor thresholds for 1 and 4 kHz sinewaves were obtained with a burst duration of 10 ms when bursts were delivered at 50 Hz (Ward & Lucas‐Toumbourou, 2007) and 30 ms when bursts were delivered at 20 Hz (Ward & Chuen, 2009). However, these findings contrast with Kantor et al. (1994) who stimulated the wrist extensors and ankle dorsiflexors with bursts of stimulation at 50 Hz. Kantor et al. found no differences in somatosensory perception and motor thresholds when repetitive bursts of a 2.5 kHz rectangular waveform composed of 10 or 25 pulses, with burst durations of 4 or 10 ms, respectively, were compared to a single pulse delivered at 50 Hz. Aside from the difference in waveform shape, it is not clear why there is a discrepancy in findings between studies. Moreover, any summation of kilohertz‐frequency waveforms on the nerve fibre membrane would be difficult to determine in these studies as they stimulated the muscles’ motor points and motor thresholds were derived from visible or palpable muscle contractions (Kantor et al., 1994; Ward & Chuen, 2009; Ward & Lucas‐Toumbourou, 2007) or extrapolated from submaximal tetanic force (Ward & Robertson, 2001).

Unlike motor‐point stimulation, burst‐modulated currents in transcutaneous spinal cord stimulation use a different set of waveform values to modulate spinal motor excitability, even though the stimulus likely activates peripheral nerve fibres as they enter and exit the spinal cord rather than the spinal cord directly (Danner et al., 2011; Finn et al., 2025; Ladenbauer et al., 2010). Transcutaneous spinal cord stimulation often involves 1‐ms bursts of a 10‐kHz rectangular waveform that is repeated at 30 Hz (Gad et al., 2018; Gerasimenko et al., 2015; Inanici et al., 2021; Parhizi et al., 2021; Sayenko et al., 2019); this equates to 10 pulses per burst of stimulation. Whether a 1‐ms burst duration is optimal for the summation of subthreshold depolarisations with nerve stimulation has not been systematically investigated. Presumably, the 1‐ms burst duration was chosen to match the typical width of a conventional pulsed current, and possibly to maximise the difference in pain and motor thresholds with electrical stimulation (e.g. Ward & Chuen, 2009; Ward & Lucas‐Toumbourou, 2007). In this regard, two studies have used shorter burst durations of 0.3–0.5 ms (Gerasimenko et al., 2015; Gorodnichev et al., 2012).

More recently, several studies have demonstrated summation for subthreshold stimuli with nerve stimulation. Formento et al. (2018) showed that, when compared to a single pulse, a burst of four pulses reduces the threshold of lower‐limb muscle responses evoked by epidural spinal cord stimulation. In the median nerve, Urriza et al. (2016) reported that a burst of 2–5 pulses at a stimulus intensity of 90% of the single‐pulse motor threshold was capable of evoking a compound muscle action potential (CMAP) in the abductor pollicis brevis muscle. However, both of these studies stimulated at 500 Hz or less, with inter‐stimulus intervals longer than 1 ms. For transcutaneous spinal cord stimulation at 10 kHz, the strength–duration curve in Dalrymple et al. (2023) indicates that the threshold for spinally‐evoked muscle responses declines as burst duration increases from 0.1 to 2 ms. An optimal burst duration was not investigated. Moreover, with transcutaneous spinal cord stimulation, it is unclear whether summation would be due to the activation of posterior (sensory) or anterior (motor) root fibres, or both (Danner et al., 2011; Ladenbauer et al., 2010), and therefore may be influenced by other spinal and supraspinal interactions (Knikou, 2008; Zehr, 2002).

The purpose of the present study was to determine the effect of the number of pulses per burst of stimulation for a 10‐kHz rectangular waveform on the activation of sensory and motor axons. Here, a single burst of stimulation was delivered to the ulnar nerve at each stimulus intensity. We hypothesised that increasing the number of pulses from 1 to 10 would decrease the current amplitude at the motor threshold for CMAPs in the abductor digiti minimi muscle as well as reduce the current required to produce a maximal CMAP (*M*_max_). Similarly, we expected that less current would be required to evoke sensory nerve action potentials (SNAPs) in digital nerves as the number of pulses increased. As an indirect measure of summation, onset latencies for CMAPs were compared across the number of pulses. The stimulus intensities at motor threshold and *M*_max_ were also expressed in terms of total phase charge because the number of pulses alters the total charge at a given current amplitude. As pulse width (Bostock, 1983; Mogyoros et al., 1996) or burst duration (Dalrymple et al., 2023) increase, the strength–duration curve for current decreases, whereas the charge–duration curve increases.

## 2 METHODS

### 2.1 Ethical approval

Twelve healthy abled‐bodied adults (seven females) with mean age of 37.7 years (SD 7.5) were recruited. Participants had no neuromuscular injuries or disorders. All procedures were approved by the University of New South Wales Human Research Ethics Committee (Reference: HC190731). The experimental protocol was explained in detail and informed consent was obtained in writing. These data formed the second part of a larger study on pain tolerance with burst‐modulated currents (Luu et al., 2024) and were collected on the same day as the pain tolerance study, for each participant, using the same experimental set‐up. This study conformed to the standards set by the *Declaration of Helsinki* (2013), except for registration in a public database (clause 35).

### 2.2 Experimental set‐up

**FIGURE 1:**
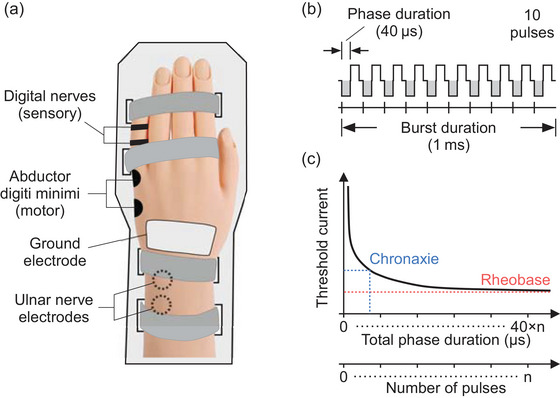
Experimental set‐up and stimulus parameters. (a) The left hand was secured in a splint made from 12‐mm‐thick high‐density foam affixed to a 6‐mm‐thick plastic backing sheet. The ulnar nerve was stimulated transcutaneously at the wrist. Surface electrodes recorded sensory nerve action potentials from digital nerves and compound muscle action potentials from the abductor digiti minimi muscle. (b) Stimulus waveform composed of 10 biphasic rectangular pulses. Vertical bars underneath each pulse indicate the triggering times of the stimulator at 10 kHz. (c) Features of a strength–duration curve for bursts of stimulation with *n* number of pulses and phase durations of 40 µs. The *x*‐axis represents the total phase duration for each burst of stimulation, but it can also be equated to the number of pulses in each burst of stimulation. The rheobase (red) is the minimal current required to produce an action potential for a stimulus of infinite duration. The chronaxie (blue) represents the stimulus duration that corresponds to two times the rheobase current.

The experimental set‐up has been described in detail previously (Luu et al., 2024). Briefly, participants were seated in a chair with their hands pronated and resting on a pillow placed across the thighs. The left hand was held in a splint to prevent flexion of the wrist during stimulation (Figure 1a). The skin was checked for damage and then cleaned and abraded with gauze soaked in 70% ethanol (w/w) solution. To record CMAPs with electromyography, surface Ag–AgCl electrodes (Cleartrace, ConMed Corp., Utica, NY, USA) were placed over the belly of the abductor digiti minimi muscle (active) and on the ulnar aspect of the fifth metacarpal phalangeal joint of the left hand (reference). To record SNAPs, ring electrodes (Ag–AgCl, The Electrode Store, Buckley, WA, USA) were placed around the midpoint of the proximal phalanx (active) and the distal interphalangeal joint (reference) of the fifth digit to record from the digital nerves. A common ground electrode (1180, 3M Health Care, St Paul, MN, USA) was trimmed to fit on the dorsum of the wrist. As the abductor digiti minimi muscle and the skin overlying the fifth digit are solely innervated by the ulnar nerve, CMAPs and SNAPs were recorded simultaneously to avoid repetition of the protocol for sensory and motor responses.

The left ulnar nerve was stimulated transcutaneously at the wrist with a constant‐current stimulator (DS8R, Digitimer Ltd, Welwyn Garden City, UK). The stimulator delivered a biphasic rectangular pulse and its firmware was modified to allow triggering at 10 kHz. However, the maximum phase duration permitted by the stimulator when triggering at 10 kHz was 40 µs due to a minimum interphase interval of 1 µs and a minimum change in phase duration of 10 µs. While a phase duration of 40 µs corresponds to a frequency of 12.5 kHz for biphasic pulses, in the present study, we refer to the stimulus waveform's carrier frequency as 10 kHz as pulses in each burst of stimulation were triggered at 10 kHz (Figure 1b). A bar electrode (inter‐electrode distance: 30 mm, Spes Medica, Battipaglia, Italy) with conductive gel (Signal gel, Parker Laboratories, Inc., Fairfield, NJ, USA) was used to probe the optimal site for stimulation. The bar electrode was initially positioned along the ulnar nerve ∼40 mm proximal to the pisiform. It was then repositioned to find the location with the largest peak‐to‐peak amplitude in the CMAP for a single pulse with a fixed stimulus intensity of 3 or 4 mA. The probing stimulus used a phase duration of 400 µs, which was reset to 40 µs for all experimental conditions. The optimal site for stimulation was then cleaned and the bar electrode was replaced with two surface electrodes (Cleartrace, ConMed Corp.) with the cathode distal for the first phase of the waveform.

Data were acquired with a Power 1401 acquisition system (Cambridge Electronic Design, Cambridge, UK) and controlled by a computer with Spike2 software (v7.12, Cambridge Electronic Design). Recordings from abductor digiti minimi were band‐pass filtered at 10–2000 Hz and amplified ×300 (1902 pre‐amplifier, Cambridge Electronic Design). Recordings from the digital nerves were passed into a Hum Bug device (Quest Scientific, North Vancouver, Canada) to remove mains noise online and then band‐pass filtered at 20–2000 Hz and amplified ×10,000 for one participant, × 3000 for 9 of 12 participants, and ×1000 in the remaining two participants (1902 pre‐amplifier, Cambridge Electronic Design). An analogue signal of current amplitude was sampled at 100 Hz. All other recordings were sampled at 10,000 Hz.

### 2.3 Protocol

Participants were asked to remain relaxed while the ulnar nerve was stimulated. Recordings from abductor digiti minimi were monitored by the experimenter for electromyographic activity. A total of six trials were performed in random order with the stimulus waveform composed of 1, 2, 4, 6, 8 or 10 pulses at 10 kHz. These corresponded to burst durations of 0.081, 0.181, 0.381, 0.581, 0.781 and 0.981 ms, respectively; however, for consistency with stimulation at 10 kHz, they are herein referred to as 0.1, 0.2, 0.4, 0.6, 0.8 and 1 ms. For each trial, participants received a single burst of stimulation about every 3 s, with stimulus intensity increasing with successive bursts. Current amplitude was increased in 1‐mA increments from 1 mA up until motor threshold, and then by 3–5 mA up to *M*_max_. A short rest period was provided between trials if participants experienced any residual sensations from stimulation.

### 2.4 Data analysis

Data from one participant were excluded from analysis as this person experienced discomfort from the stimulation after one trial. In subsequent trials, the participant was not able to tolerate stimulus intensities above 22% of the *M*_max_ recorded from the accompanying earlier experiment on pain tolerance (Luu et al., 2024).

The motor threshold for each stimulus waveform was determined by visual inspection as the minimal current required to produce a CMAP in the electromyographic recordings. The size of the CMAP was measured from its peak‐to‐peak amplitude. The minimal current that produced *M*_max_ was measured at the first CMAP where the increase in amplitude plateaued and was less than 2% for consecutive increases in stimulus intensity. Total phase charge at motor threshold and *M*_max_ were calculated as current (mA) × number of pulses × phase duration (40 µs), and are expressed in units of microcoulombs (µC). The efficacy of each stimulus waveform at *M*_max_ was determined by dividing CMAP amplitude by total phase charge.

Onset latencies for CMAPs were determined at motor threshold for one participant and at low‐level suprathreshold intensities for all other participants, due to inconsistencies in the shape of the potential at motor threshold across the six stimulus waveforms. A consistent potential shape was evoked at stimulus intensities of 1.1–2.0 times motor threshold, which corresponded to CMAP amplitudes of 0.5–2% of *M*_max_. Onset latencies were determined by visual inspection as the time of the first deflection in CMAP amplitude from baseline relative to the time of the first pulse in each burst of stimulation.

The rheobase current and chronaxie of motor axons were determined for each participant from the linear fit of the charge–duration curve as described in Bostock (1983) and Mogyoros et al. (1996). Rheobase current for the CMAP is defined as the minimal current required to elicit an action potential with a waveform of infinite duration. The chronaxie is the total phase duration at two times the rheobase current and can be equated with the time constant of decay for the strength–duration relationship (Figure 1c). Charge was taken as the total phase charge and duration was taken as the total phase duration for each waveform from 1 up to 10 pulses. The slope of the linear fit corresponded to the rheobase current and the absolute value of the *x*‐intercept corresponded to the chronaxie.

Sensory nerve recordings were filtered offline with a zero‐phase, fourth‐order high‐pass Butterworth filter with a cut‐off frequency of 400 Hz to remove the tail of the stimulus artefact. The size of the SNAP was measured from its peak‐to‐peak amplitude. However, since SNAPs were recorded simultaneously with CMAPs, maximal SNAPs were obtained in only 3 of 11 participants before stimulation was terminated at the stimulus intensity for *M*_max_. Therefore, individual stimulus–response curves from these three participants are shown to illustrate the effect of the number of pulses over the full range of SNAP amplitudes. Sensory thresholds – the minimal current required to produce a SNAP in digital nerve recordings – were determined for all participants by visual inspection. Note that in the present study, the sensory threshold determined from a single SNAP may be less accurate than the motor threshold due to the smaller signal‐to‐noise ratio of SNAPs compared to CMAPs. Additionally, sensory threshold was measured when increments in stimulus intensity changed from steps of 1 mA to 3–5 mA due to our prioritisation of motor recruitment. Total phase charge at sensory threshold was calculated in the same manner as for motor threshold: current (mA) × number of pulses × phase duration (40 µs), and are expressed in units of microcoulombs (µC).

### 2.5 Statistical analysis

Statistical analyses were performed in R (version 4.3.0, R Foundation for Statistical Computing, Vienna, Austria) using the *lmerTest* and *emmeans* packages for mixed linear models fit by restricted maximum likelihood estimation. Random intercepts were included for participants in all models. The effect of the number of pulses in each burst of stimulation on motor threshold was determined with separate mixed linear models for current amplitude and total charge. Planned contrasts for consecutive increases in the number of pulses were made with adjustments using the multivariate *t* distribution. The same parameters were used in separate mixed linear models for onset latency, current amplitude and total phase charge at *M*_max_, CMAP amplitude of *M*_max_, and waveform efficacy. For onset latency, estimated marginal means were also compared with Tukey's method for pairwise comparisons. The difference in chronaxies at motor threshold and *M*_max_ was determined with Student's paired‐samples *t*‐test. The effect of the number of pulses in each burst of stimulation on sensory threshold was determined with separate mixed linear models for current amplitude and total charge. For sensory threshold, planned contrasts for consecutive increases in the number of pulses were made with adjustments using the multivariate *t* distribution. Group results presented in figures are descriptive means with 95% confidence intervals. Results in the text are estimated marginal means, except for rheobase currents and chronaxies, which are descriptive means, with 95% confidence intervals. Statistical significance was set at *P* < 0.05.

## 3 RESULTS

**FIGURE 2:**
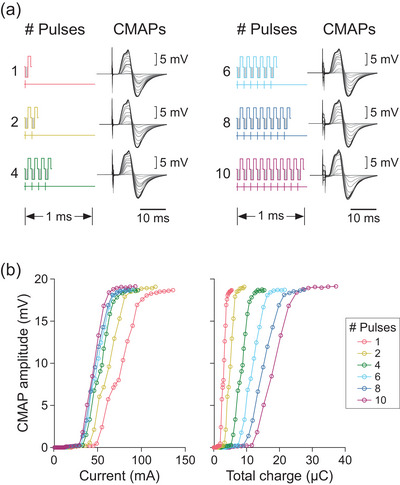
Stimulus waveforms and data from an individual participant. (a) The six stimulus waveforms, each composed of a different number (#) of pulses, are shown in separate colours. Raw data for the compound muscle action potentials (CMAPs) are superimposed for each burst of stimulation from 1 mA up to the maximal CMAP, and are immediately to the right of the corresponding waveform. Vertical bars below each waveform indicate the triggering times of the stimulator. (b) Stimulus–response curves for CMAP amplitudes are plotted against current (left) and total phase charge (right) for each burst of stimulation. The same colours for the number of pulses has been retained for Figures 3, 4, 5, 7, 7 and 8.

Data from an individual participant in Figure 2a show that the different number of pulses produced CMAPs of similar shape and peak‐to‐peak amplitude as stimulus intensity was increased up to *M*_max_. For this participant, increasing the number of pulses in the stimulus waveform reduced the current needed to activate motor axons in the ulnar nerve, as indicated by the progressive shift in the stimulus–response curve to the left (left panel, Figure 2b). The magnitude of these shifts in current diminished as the number of pulses increased. However, when stimulus intensity was expressed in terms of the total phase charge per burst of stimulation, more charge was required to activate the motor axons as the number of pulses increased, as indicated by the right‐ward shift in the stimulus–response curve (right panel, Figure 2b). Notably, as the number of stimulus pulses increased, the stimulus–response curves plotted against total phase charge shifted in reverse order relative to those plotted against current.

**FIGURE 3:**
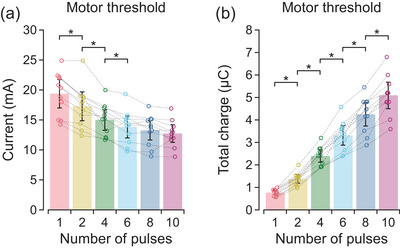
Motor threshold. Values are descriptive means and 95% confidence intervals of all 11 participants. Data from each individual participant (circles) are connected by a dashed line. The number of pulses in the stimulus waveform is shown in a separate colour. At motor threshold, the current (a) decreased while the total phase charge (b) increased with the addition of more pulses to the stimulus waveform. Asterisks (*) indicate a statistically significant difference between consecutive increases in the number of pulses as determined by planned contrasts.

As shown in Figure 3a, increasing the number of pulses in the stimulus waveform decreased the current at motor threshold (*F*_5,50_ = 71.2, *P* < 0.001). The single‐pulse motor threshold of 19.4 [17.5, 21.3] mA decreased gradually as the number of pulses increased up to six pulses (Table 1). At six pulses, the current amplitude was reduced to 71.4% [64.8, 78.0%] of the single‐pulse motor threshold. Figure 3b shows the opposite behaviour for total charge at motor threshold, which increased with the number of pulses (*F*_5,50_ = 283.5, *P* < 0.001). The total charge of 0.78 [0.40, 1.15] µC for the single‐pulse motor threshold increased progressively as the number of pulses increased up to the maximum of 10 pulses (Table 1).

**TABLE 1:** Changes at motor threshold for consecutive increases in the number of pulses.

Change in number of pulses	Current (% change)	Total charge (% change)	Onset latency^†^ (% change)	CMAP amplitude^†^ (% change)
1–2 pulses	−10.8 [−16.6, −4.9]	78.3 [30.8, 125.9]	3.3 [−5.9, 12.5]	−1.4 [−20.7, 18.0]
2–4 pulses	−13.1 [−19.7, −6.5]	73.7 [47.0, 100.3]	5.5 [−3.4, 14.4]	0.9 [−18.7, 20.6]
4–6 pulses	−7.9 [−15.5, −0.3]	38.2 [22.8, 53.5]	4.8 [−3.6, 13.3]	−0.2 [−19.7, 19.3]
6–8 pulses	−3.9 [−12.2, 4.3]	28.1 [17.0, 39.2]	3.5 [−4.6, 11.6]	−2.7 [−22.2, 16.8]
8–10 pulses	−4.1 [−12.7, 4.5]	19.9 [11.2, 28.5]	4.5 [−3.4, 12.3]	−10.6 [−30.6, 9.5]

*Note*: Estimated marginal means and 95% confidence intervals. A negative change represents a decrease in motor threshold as the number of pulses increased. †Measures at up to two times motor threshold (see Methods) for potential amplitudes of 0.5–2% of the maximal compound muscle action potential (CMAP).

**FIGURE 4:**
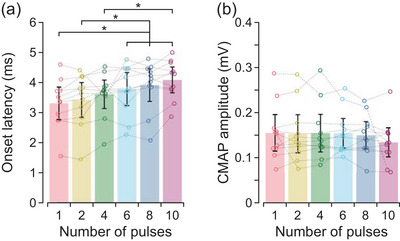
Onset latencies at low‐level suprathreshold intensities. Values are descriptive means and 95% confidence intervals of all 11 participants. Data from each individual participant (circles) are connected by a dashed line. The number of pulses in the stimulus waveform is shown in a separate colour. (a) At stimulus intensities of up to two times motor threshold, onset latencies increased as more pulses were added to the stimulus waveform. (b) The compound muscle action potential (CMAP) amplitudes from which onset latencies were measured. Asterisk (*) indicates a statistically significant difference as determined by pairwise comparisons with Tukey's method.

Onset latency increased with the number of pulses (*F*_5,50_ = 13.2, *P* < 0.001), but there were no statistically significant differences in latencies for consecutive increases in the number of pulses (Table 1). Rather, when compared to the latency of 3.3 [2.8, 3.8] ms for a single pulse, onset latency increased by 0.47 [0.13, 0.82] ms for six pulses, 0.61 [0.26, 0.95] ms for eight pulses, and 0.78 [0.44, 1.12] ms for 10 pulses (Figure 4a). There was no effect (*F*_5,50_ = 1.0, *P* = 0.425) of number of pulses on CMAP amplitudes from which onset latencies were measured (Figure 4b).

**FIGURE 5:**
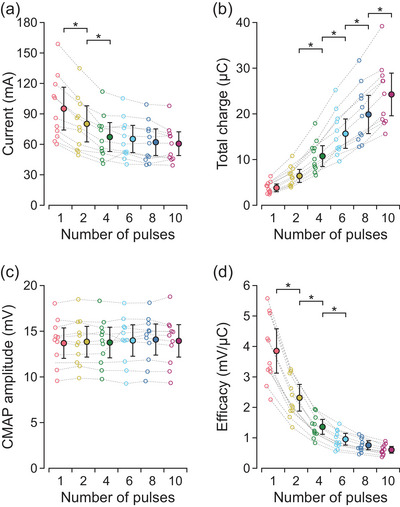
Maximal compound muscle action potential (CMAP). Values are descriptive means (filled circles) and 95% confidence intervals of all 11 participants. Data from each individual participant (open circles) are connected by a dashed line. The number of pulses in the stimulus waveform is shown in a separate colour. At the maximal CMAP, the current (a) decreased while the total phase charge (b) increased with the addition of more pulses; but there were no statistically significant differences in CMAP amplitudes (c) between stimulus waveforms. The efficacy of each waveform at the maximal CMAP was calculated by dividing the CMAP amplitude by the total phase charge for each participant (d). Asterisks (*) indicate a statistically significant difference between consecutive increases in the number of pulses as determined by planned contrasts.

The pattern of decrease in current (*F*_5,50_ = 46.7, *P* < 0.001, Figure 5a) and increase in total charge (*F*_5,50_ = 123.8, *P* < 0.001, Figure 5b) as the number of pulses increased was maintained at *M*_max_. The minimal current of 95.2 [79.9, 110.5] mA at the single‐pulse *M*_max_ decreased gradually as the number of pulses increased up to four pulses (Table 2). At four pulses, the current amplitude was reduced to 70.6% [62.0, 79.2%] of the single‐pulse intensity. There was no statistically significant difference in total charge of 3.8 [0.9, 6.8] µC for the single‐pulse *M*_max_ and 6.4 [3.5, 9.4] µC for the two‐pulse *M*_max_. However, total charge at *M*_max_ increased progressively as the number of pulses increased from two up to the maximum of 10 pulses (Table 2).

**TABLE 2:** Changes at the maximal compound muscle action potential (CMAP) for consecutive increases in the number of pulses.

Change in number of pulses	CMAP amplitude (% change)	Current (% change)	Total charge (% change)	Efficacy (% change)
1–2 pulses	1.1 [−3.0, 5.2]	−15.8 [−23.4, −8.1]	68.5 [−1.0, 137.8]	−39.9 [−50.2, −29.6]
2–4 pulses	−0.6 [−4.7, 3.5]	−16.2 [−25.3, −7.1]	67.6 [26.4, 108.9]	−41.3 [−58.4, −24.0]
4–6 pulses	−1.6 [−2.5, 5.7]	−2.8 [−13.7, 8.0]	45.8 [21.1, 70.3]	−29.9 [−59.2, −0.6]
6–8 pulses	0.8 [−3.3, 4.8]	−4.9 [−16.1, 6.3]	26.8 [9.9, 43.7]	−20.6 [−62.5, 21.1]
8–10 pulses	−1.0 [−5.0, 3.0]	−2.3 [−14.1, 9.4]	22.1 [8.8, 35.4]	−19.9 [−72.7, 32.7]

*Note*: Estimated marginal means and 95% confidence intervals. A negative change represents a decrease in amplitude, threshold, or a loss in efficacy as the number of pulses increased.

Figure 5c shows that there was no effect of number of pulses on CMAP amplitudes at *M*_max_ (*F*_5,50_ = 0.93, *P* = 0.470). As a result, the efficacy in producing an *M*_max_ decreased (*F*_5,50_ = 135.6, *P* < 0.001) as the number of pulses increased (Figure 5d). The efficacy of 3.9 [3.5, 4.2] mV/µC for a single pulse at *M*_max_ decreased gradually as the number of pulses increased up to six pulses (Table 2).

**FIGURE 6:**
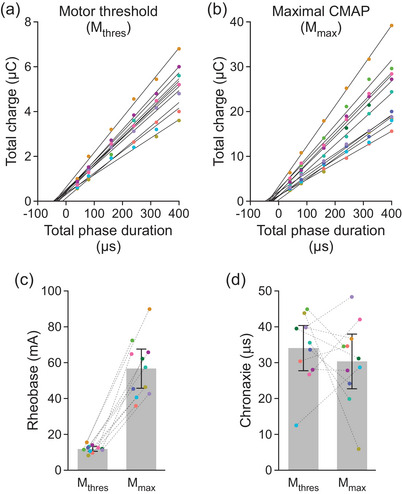
Charge–duration curves. Data from each individual participant (dots) are shown in the same colour in a–d. Data in (a) and (b) are reproduced from Figures 3b and 5b, respectively, but shown here on a continuous scale for the *x*‐axis. Total phase duration was calculated as 40 µs × number of pulses, as described in Figure 1c. Mean values in (c, d) are descriptive means and 95% confidence intervals of all 11 participants. The linear best fits (continuous lines) for each participant had a mean *R* ^2^ of 0.994 [0.990, 0.997] at motor threshold (*M*_thres_, a) and 0.988 [0.981, 0.995] at the maximal compound muscle action potential (*M*_max_, b). Rheobase currents (c) are derived from the slopes and chronaxies (d) are derived from the *x*‐intercepts of the linear fits of the charge–duration curves. In (c, d) data from each individual participant are connected by a dashed line. CMAP, compound muscle action potential.

Linear fits of the charge–duration curves for motor threshold are shown in Figure 6a, and those for *M*_max_ are shown in Figure 6b. Rheobase current was 11.9 [10.6, 13.3] mA at motor threshold and 56.7 [45.7, 67.7] mA at *M*_max_ (Figure 6c). There was no statistically significant difference (*t* = −0.785, *P* = 0.445) between chronaxies of 34.1 [27.8, 40.4] µs at motor threshold and 30.3 [22.7, 39.0] µs at *M*_max_ (Figure 6d).

**FIGURE 7:**
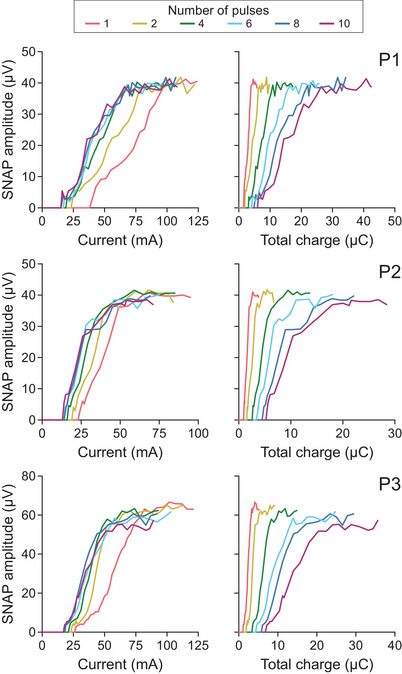
Sensory nerve action potentials (SNAPs). Stimulus–response curves are shown for the three participants (P1, P2 and P3) in whom we obtained the full range of SNAPs from sensory threshold up to the maximal SNAP. Potential amplitudes are plotted against current (left) and total phase charge (right) for each burst of stimulation. The six stimulus waveforms, each composed of a different number of pulses, are shown in separate colours.

For SNAPs, as the number of pulses increased, the stimulus–response curves were shifted to the left when plotted for current (left column, Figure 7), and to the right when plotted for total charge (right column, Figure 7). These shifts in the stimulus–response curves for SNAPs were consistent with the pattern for CMAPs in Figure 2b.

**FIGURE 8:**
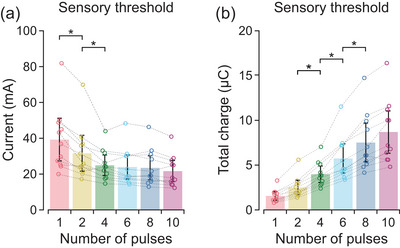
Sensory threshold. Values are descriptive means and 95% confidence intervals of all 11 participants. Data from each individual participant (circles) are connected by a dashed line. The number of pulses in the stimulus waveform is shown in a separate colour. At sensory threshold, the current (a) decreased while the total phase charge (b) increased with the addition of more pulses to the stimulus waveform. Asterisks (*) indicate a statistically significant difference between consecutive increases in the number of pulses as determined by planned contrasts.

The current at sensory threshold decreased as the number of pulses in the stimulus waveform increased (*F*_5,50_ = 24.4, *P* < 0.001). The sensory threshold for a single pulse was 39.3 [31.3, 47.2] mA, which decreased gradually to 25.0 [17.1, 32.9] mA as the number of pulses increased up to four pulses (Figure 8a). The total charge at sensory threshold increased with the number of pulses (*F*_5,50_ = 58.8, *P* < 0.001). However, there was no statistically significant difference in total charge between the single‐pulse and two‐pulse waveforms (Figure 8b). The total charge at sensory threshold increased progressively as the number of pulses increased from two to eight.

## 4 DISCUSSION

The present study examined the effect of the number of pulses per burst of stimulation on the activation of sensory and motor axons in the ulnar nerve. For a 10‐kHz biphasic waveform, the increase in number of pulses shifted the CMAP and SNAP stimulus–response curves for current to the left, which means less current was required to activate sensory and motor axons. Accordingly, the current amplitudes at sensory threshold and *M*_max_ gradually decreased as the number of pulses increased from one up to four pulses, whereas motor threshold decreased gradually from one up to six pulses. Conversely, the addition of more pulses increased the total phase duration, shifting the CMAP and SNAP stimulus–response curves for charge to the right, with the shift sequence reversed compared to those for current. At motor threshold and *M*_max_, the total charge progressively increased with the number of pulses from one and two pulses, respectively, up to the maximum of 10 pulses. Total charge at sensory threshold progressively increased from two up to eight pulses. As there were no statistically significant differences in CMAP amplitudes at *M*_max_, the increase in total charge with the number of pulses reduced efficacy at *M*_max_ from one up to six pulses. When compared to a single pulse, onset latencies were delayed for stimulus waveforms with six or more pulses. These findings provide evidence for a Gildemeister effect in sensory and motor axons in humans.

Our results for ulnar‐nerve stimulation are consistent with those of Ward and colleagues (Ward & Chuen, 2009; Ward & Lucas‐Toumbourou, 2007) for motor‐point stimulation of the wrist extensors in that the motor threshold for current decreased as the number of pulses increased. However, our 10 kHz waveform showed this decrease in motor threshold over a shorter burst duration of 0.6 ms (six pulses) than the 10 and 30 ms reported by Ward and colleagues. Notably, the 1‐ and 4‐kHz carrier waveforms used in their studies both had optimal burst durations of 10 ms when bursts of stimulation were repeated at 50 Hz (Ward & Lucas‐Toumbourou, 2007) and 30 ms when bursts were repeated at 20 Hz (Ward & Chuen, 2009). This suggests that their changes in motor threshold depended more on repetition frequency than on the number of pulses in each burst of stimulation. Since our study only used single bursts of stimulation, we cannot make any comparisons with regard to repetition frequency. Nevertheless, other factors contributed to the differences in burst duration between studies. We measured motor threshold from the CMAP, while Ward and colleagues relied on less sensitive visible contractions under the skin or movement of the joint. Therefore, it is likely that their motor thresholds are at a higher contraction intensity and reflect not only the summation of depolarised membrane potentials but also the summation of twitch forces, particularly with repetition frequencies of 20 and 50 Hz (e.g. Bigland‐Ritchie et al., 1979; Thomas et al., 1991). Additionally, Ward and colleagues reported a quadratic trend in the data and determined an optimal burst duration based on the minimum motor threshold at the vertex, rather than comparing motor thresholds for consecutive increases in burst duration.

Paired‐pulse paradigms, such as those used in the method of latent addition (Bostock & Rothwell, 1997; Panizza et al., 1994) and to investigate the phenomenon of subthreshold superexcitability (Bostock et al., 2005), provide insights into the time course for summation of subthreshold depolarisations of the axonal membrane. Both paradigms use a subthreshold conditioning pulse to modulate the threshold of a subsequent test pulse. In the ulnar nerve, latent addition induced by a 50–60 µs conditioning pulse at 90% of the unconditioned threshold causes a decrease in threshold that can last up to one millisecond, with an exponential time constant of recovery of 145–200 µs for motor axons and 339–367 µs for sensory axons (Bostock & Rothwell, 1997; Panizza et al., 1994). The increases in sensory and motor excitability persists for longer than the 100 µs inter‐pulse interval of our 10‐kHz waveform and are largely attributed to the passive properties of the membrane time constant (Panizza et al., 1994), although local activation of sodium channels also contributes (Bostock & Rothwell, 1997). These active and passive components of latent addition are also thought to explain subthreshold superexcitability in single motor axons of the median and ulnar nerves (Bostock et al., 2005). Here, a conditioning pulse of 1 ms duration at 96% of the unconditioned motor threshold leads to a reduction in threshold that can last up to tens of milliseconds; mostly due to the greater pulse charge from a wider conditioning pulse. The time courses for both latent addition and subthreshold superexcitability were determined with monophasic pulses. For biphasic waveforms, the time courses are likely to be shorter, as it appears only the active component contributes to the increase in motor excitability for charge‐balanced waveforms (Bostock et al., 2005).

While the Gildemeister effect describes the summation of subthreshold depolarisations at sensory or motor threshold, we also observed summation at *M*_max_, where current amplitudes decreased as the number of pulses increased from one up to four. Ward & Robertson (2001) postulated that summation of subthreshold depolarisations can occur at suprathreshold intensities. Once an action potential is evoked by the first pulse, subsequent pulses could further depolarise other higher‐threshold axons, resulting in asynchronous activation of motor fibres. However, in our study, efficacy at *M*_max_ decreased with more pulses, which indicates that multiple pulses are less effective than a single pulse (Figure 5d). This loss in efficacy may reflect the axon's strength–duration properties and the strength of the ‘conditioning’ pulses in each burst of stimulation. At *M*_max_, a chronaxie of 30.3 µs indicates that the 40 µs phase duration for our single pulse is already longer than the time constant of decay for the strength–duration relationship. Thus, further increases in burst duration, or the addition of more pulses, results in diminishing gains as current approaches rheobase. In regards to the strength of the conditioning pulses, a decrease in subthreshold current can be a double‐edged sword as it weakens depolarisation and limits summation time (e.g. Panizza et al., 1994). This was evident after six pulses at motor threshold and after four pulses at *M*_max_ when current decreased to 71% of the single‐pulse values. For sensory threshold, current decreased to 64% of the single‐pulse value after four pulses. Another possibility is that the hyperpolarisation phases of our biphasic waveform hindered effective summation. However, a Gildemeister‐like effect has been demonstrated for spinally‐evoked muscle responses with a monophasic 10 kHz waveform where the relative decreases in motor threshold for one up to five pulses are comparable to ours (Dalrymple et al., 2023). When the stimulus waveform in the present study comprised more than six pulses, the charge contributed by each additional pulse became an increasingly smaller percentage of the total charge and no longer influenced efficacy.

The increase in onset latency of the CMAP with the number of pulses provides further support for summation of subthreshold depolarisations. When compared to a single pulse, the onset latency for six pulses was delayed by 0.47 ms, which indicates that membrane potential did not reach threshold until the summation of the last pulse, as it was delivered 0.5 ms after the first pulse. Delays in onset latencies of 0.61 ms for eight pulses and 0.78 ms for ten pulses suggest that threshold potential was reached before the last pulse, as they were delivered 0.7 ms and 0.9 ms, respectively, after the first pulse. For eight and ten pulses, these discrepancies between the measured and delivered onset latencies might be related to our time resolution of 0.1 ms when sampling at 10 kHz, equivalent to the duration of a single pulse. This time resolution could also explain the lack of difference in onset latencies for consecutive increases in the number of pulses, as the addition of two pulses represents a difference of only 0.2 ms.

The implication of our findings for transcutaneous spinal cord stimulation, which often involves 1‐ms bursts of a 10‐kHz waveform (Gad et al., 2018; Gerasimenko et al., 2015; Inanici et al., 2021; Parhizi et al., 2021; Sayenko et al., 2019), is that temporal summation of subthreshold depolarisations occurs at 10 kHz in both sensory and motor axons. However, temporal summation at this frequency is not efficacious, as total charge increased with the number of pulses and the effect of summation on current intensity disappeared after four to six pulses. These findings suggest that the 1‐ms burst duration (10 pulses) typically used in transcutaneous spinal cord stimulation may deliver at least 40% more electrical stimulation than necessary to modulate spinal motor excitability. It should be noted that the present findings are limited to single bursts of stimulation, whereas transcutaneous spinal cord stimulation is normally delivered in repetitive bursts. Whether the same summation effects occur with repeated stimulation remains unclear and warrants further investigation. This is particularly relevant for suprathreshold stimulation intensities, as current thresholds may vary with repetition frequency due to intrinsic biophysical properties of the axon, such as refractoriness, superexcitability and subexcitability, which evolve over different time courses (see Bostock et al., 2005; Kiernan et al., 1996).

In summary, our study provides evidence of the summation of subthreshold depolarisations in sensory and motor axons with a 10‐kHz biphasic waveform. For both sensory and motor axons, there was a limit to the summative effects of kilohertz‐frequency stimulation as the decrease in threshold current plateaued after four to six pulses (or bursts of 0.4–0.6 ms). Whether a single pulse or the summation of four to six pulses is optimal for activation remains unclear. Certainly, when matched for charge, a single wide pulse is more effective at activating sensory and motor axons than five or 10 narrower pulses (Luu et al., 2024). Here, the single pulse had a lower total charge and greater efficacy at *M*_max_ than the waveforms with four to six pulses, but it also had a higher threshold current. What is clear from our results, though, is that at 10 kHz, more than six pulses is suboptimal for summation as there is no further decrease in threshold current, whereas total charge continues to increase.

## AUTHOR CONTRIBUTIONS

This work was conducted at Neuroscience Research Australia. Billy L. Luu, Harrison T. Finn, Terry Trinh, Simon C. Gandevia, Martin E. Héroux and Jane E. Butler conceived research. Billy L. Luu, Harrison T. Finn and Jane E. Butler designed methodology. Billy L. Luu collected and analysed data, prepared figures, and drafted the manuscript. Billy L. Luu, Harrison T. Finn, Terry Trinh, Simon C. Gandevia, Martin E. Héroux and Jane E. Butler interpreted results and critically revised the manuscript. All authors have read and approved the final version of this manuscript; agree to be accountable for all aspects of the work in ensuring that questions related to the accuracy or integrity of any part of the work are appropriately investigated and resolved; and all persons designated as authors qualify for authorship, and all those who qualify for authorship are listed.

## CONFLICT OF INTEREST

None declared.

## Data Availability

The data are included within the figures and are available from the corresponding author upon reasonable request.
